# Supplementary figures and images for: Characterisation of tetraspanins from Schistosoma haematobium and evaluation of their potential as novel diagnostic markers
Source: PLoS Negl Trop Dis. 2022 Jan 24;16(1):e0010151. doi: 10.1371/journal.pntd.0010151 (PMC8812969; doi:10.1371/journal.pntd.0010151)

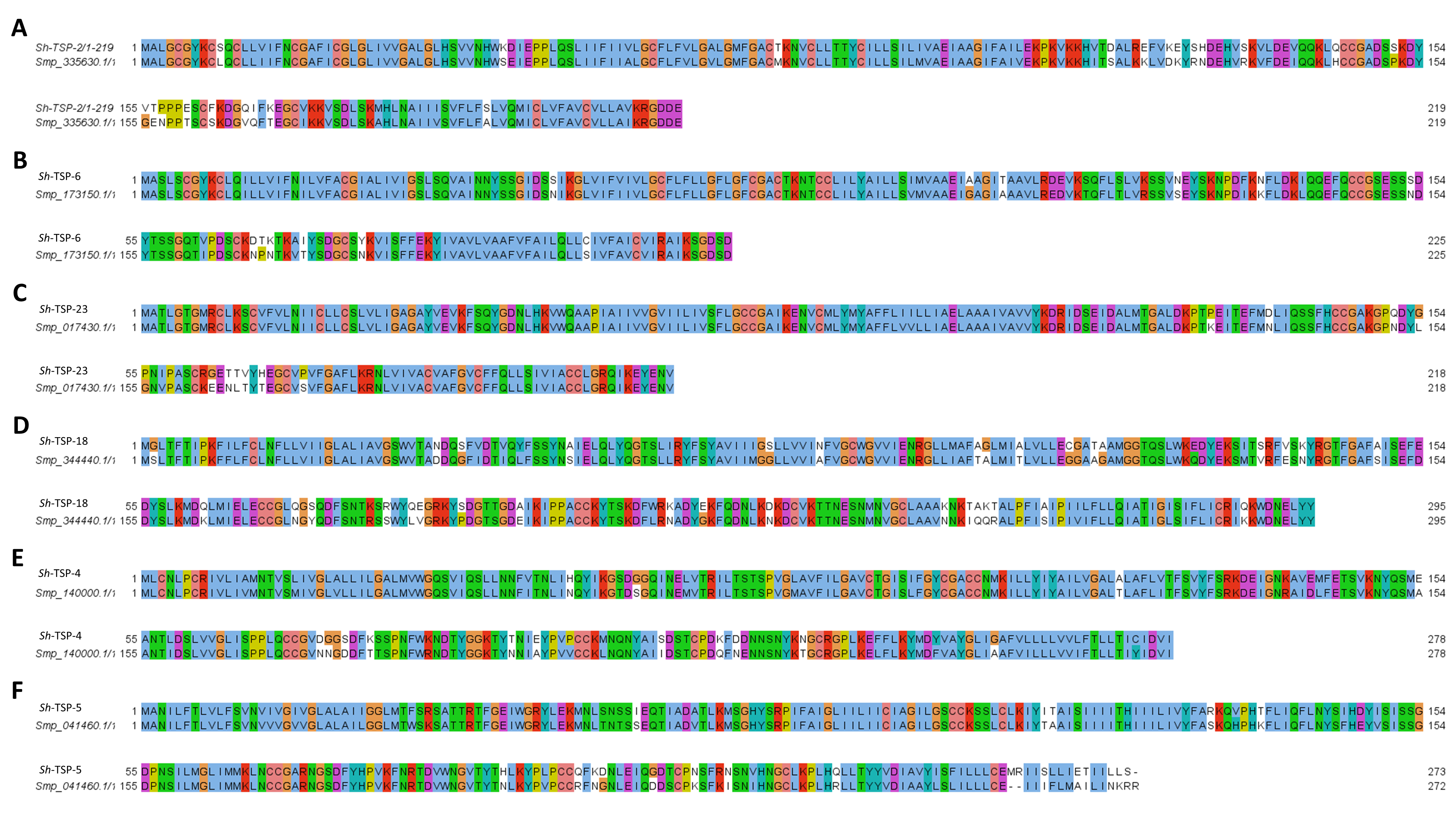

Supplement: S1 Fig — A multiple sequence alignment was carried out using MUSCLE with defaults and visualised using JalView with default ClustalX colouring. (TIF) [file pntd.0010151.s001.TIF]

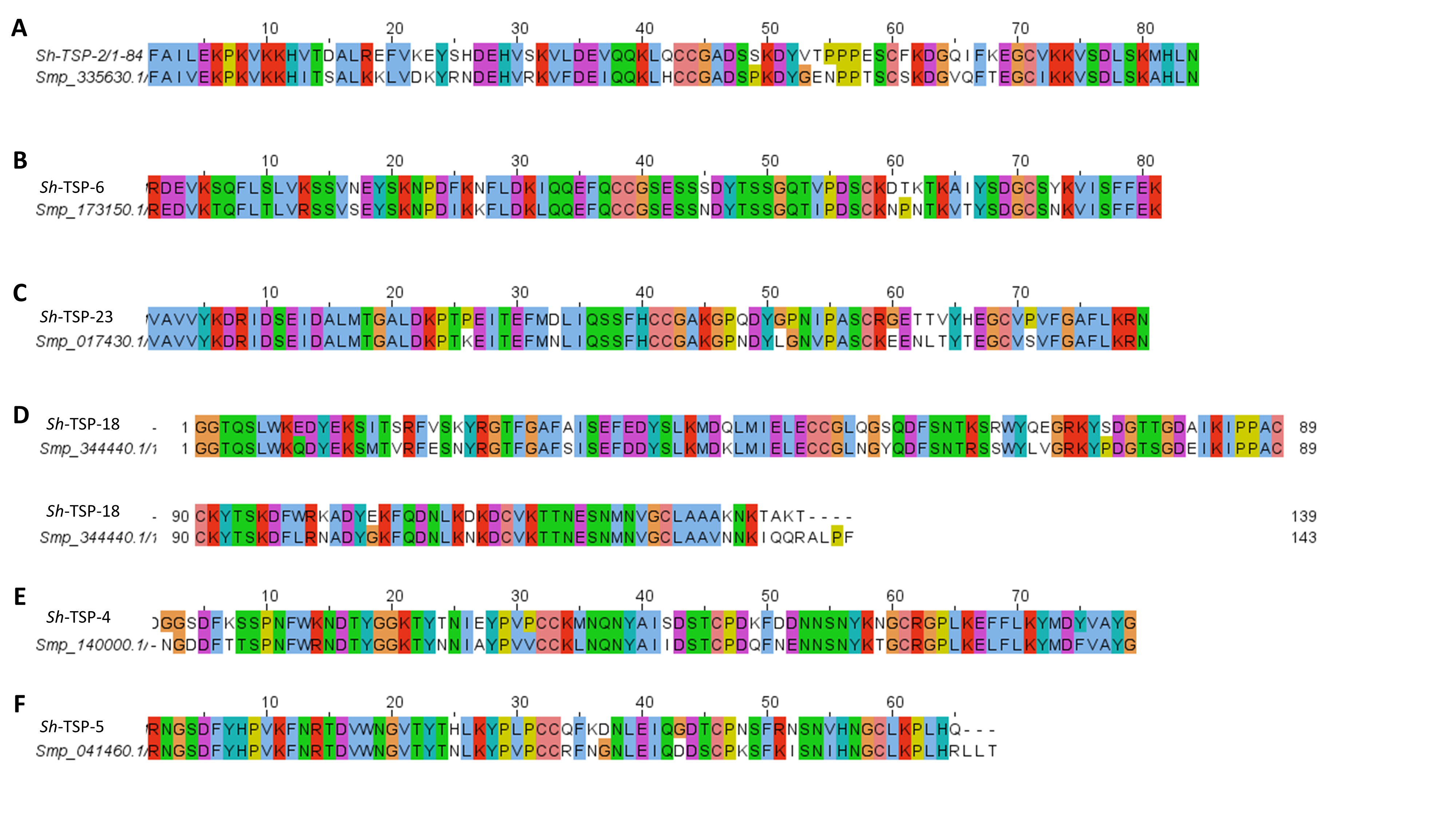

Supplement: S2 Fig — A multiple sequence alignment was carried out using MUSCLE with defaults and visualised using JalView. (TIF) [file pntd.0010151.s002.TIF]

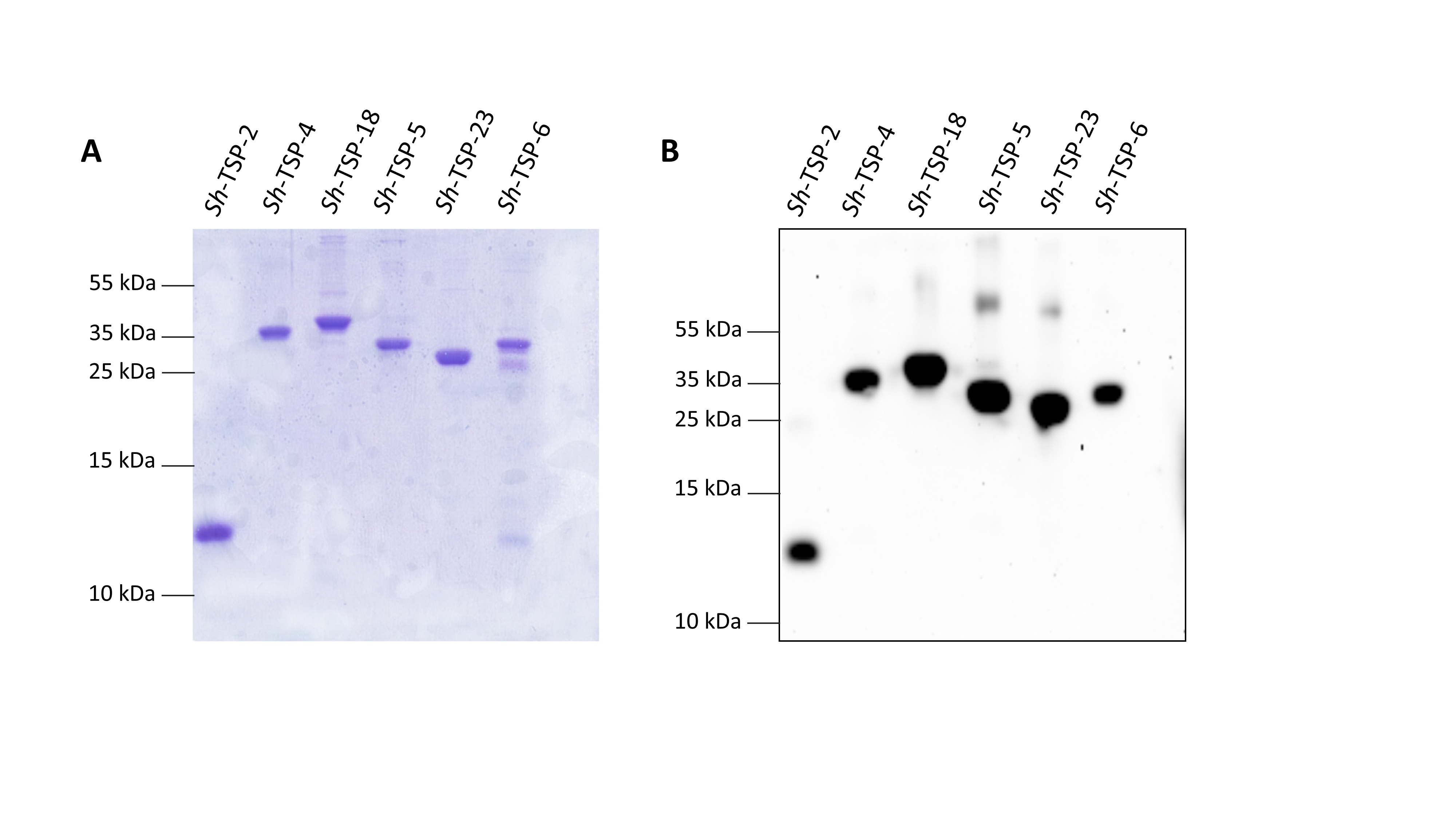

Supplement: S3 Fig — 1μg of each TSP was electrophoresed on SDS-PAGE and Comassie-stained: Protein ladder (A1), Sh-TSP-2 (A2), Sh-TSP-4 (A3), Sh-TSP-18 (A4), Sh-TSP-5 (A5), Sh-TSP-23 (A6) and Sh-TSP-6 (A7). Western blot of TSPs using an anti-His monoclonal antibody: Protein ladder (B1), Sh-TSP-2 (B2), Sh-TSP-4 (B3), Sh-TSP-18 (B4), Sh-TSP-5 (B5), Sh-TSP-23 (B6) and Sh-TSP-6 (B7). The expected size of recombinant Sh-TSP-2 was 12.4 kDa, since it was expressed without a tag. For Sh-TSP-4, Sh-TSP-5, Sh-TSP-6, Sh-TSP-18 and Sh-TSP-23, since they contained the TRX-tag from the cloning vector, their expected size ranged from ~28 kDa to ~38 kDa. (TIF) [file pntd.0010151.s003.TIF]

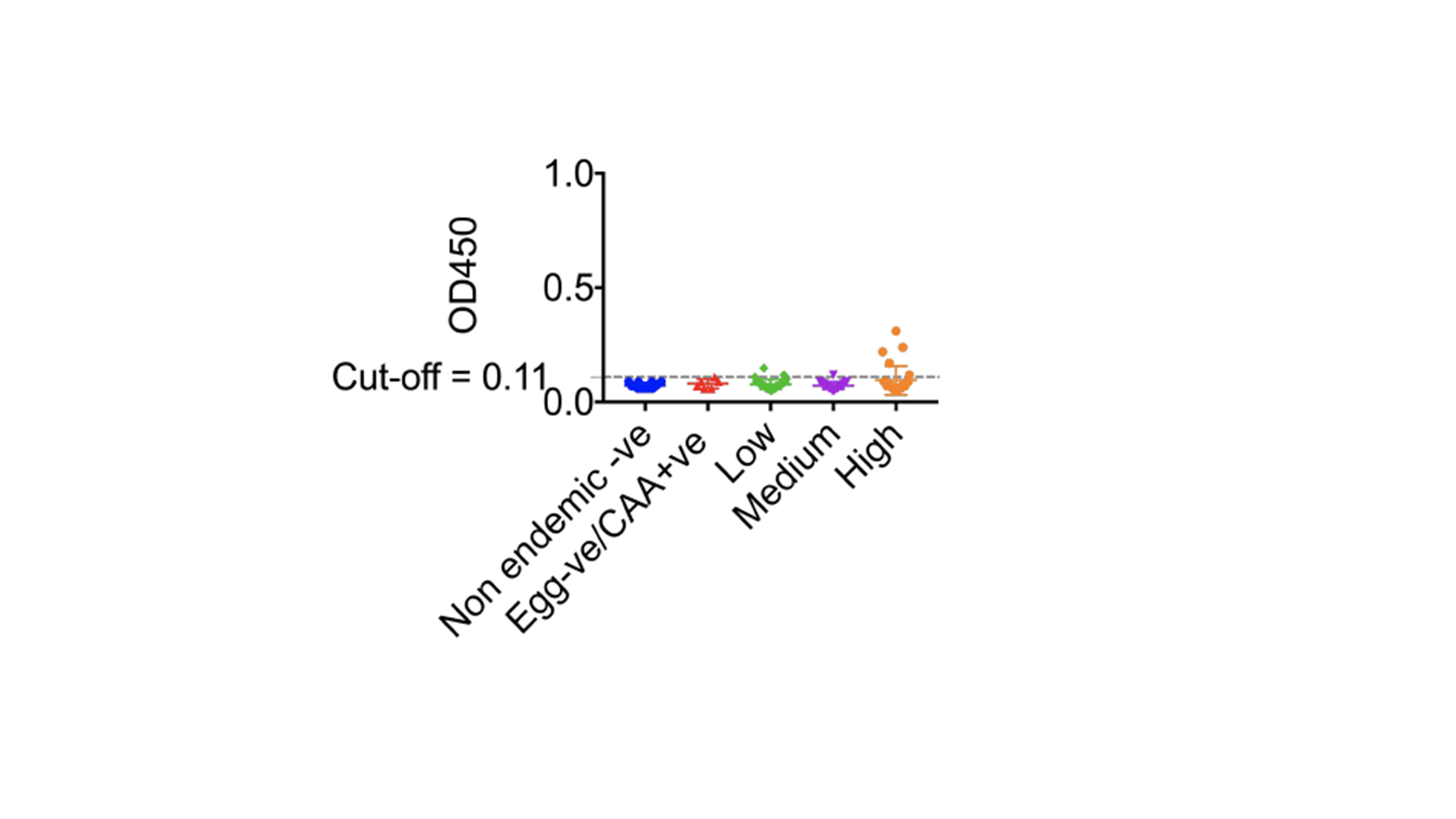

Supplement: S4 Fig — The antibody level was measured by indirect ELISA and indicated by OD values. Urine of non-infected individuals from non-endemic area was used as negative control. The reactivity cut-off values were determined by adding the average and 3x standard deviation of non-endemic negative individuals (indicated by broken lines). (TIF) [file pntd.0010151.s004.TIF]
