# Supplementary material for: Characterisation of tetraspanins from Schistosoma haematobium and evaluation of their potential as novel diagnostic markers
Source: PLoS Negl Trop Dis. 2022 Jan 24;16(1):e0010151. doi: 10.1371/journal.pntd.0010151 (PMC8812969; doi:10.1371/journal.pntd.0010151)
Supplement: S1 Table — (DOCX) [file pntd.0010151.s005.docx]

**Supplementary Table S1.** Lists of oligonucleotide primers used for qPCR analysis of *Schistosoma haematobium* tetraspanins.

| Protein | Forward primer | Reverse primer |
| --- | --- | --- |
| α-tubulin | 5´GGGCGCGTCTAGATCATAAG3´ | 5´GTCAACACCAACCTCCTCGT3´ |
| *Sh*-TSP-6 | 5´TGCTGTGCTGAGAGACGAAG3´ | 5´GGACGGTTTGTCCAGATGAT3´ |
| *Sh*-TSP-5 | 5´AGCCCGAAATGGAAGTGATT3´ | 5´TGGTTTCAAGCATCCATTATGT3´ |
| *Sh*-TSP-23 | 5´GGCCAAACATTCCAGCTTCA3´ | 5´GCGACCCAAACAACAAGCTA3´ |
| *Sh*-TSP-4 | 5´TCCTGTACCGTGTTGCAAAA3´ | 5´TGTGAATAGAACAACGAGCAACA3´ |
| *Sh*-TSP-2 | 5´CACCACCGGAATCCTGTTTC3´ | 5´CATCATCACCGCGCTTTACA3´ |
| *Sh*-TSP-18 | 5´AAATTCCTCCAGCTTGCTGT3´ | 5´TATGAACGGGAGGGCTGTTT3´ |
