# Supplementary material for: Characterisation of tetraspanins from Schistosoma haematobium and evaluation of their potential as novel diagnostic markers
Source: PLoS Negl Trop Dis. 2022 Jan 24;16(1):e0010151. doi: 10.1371/journal.pntd.0010151 (PMC8812969; doi:10.1371/journal.pntd.0010151)
Supplement: S2 Table — (DOCX) [file pntd.0010151.s006.docx]

**Supplementary Table S2.** Lists of sequences from *Bos taurus*, *Clonorchis sinensis, Danio rerio, Homo sapiens*, *Mus musculus, Opisthorchis viverrini, Schistosoma haematobium*, *Schistosoma japonicum* and *Schistosoma mansoni* used for the phylogenetic analysis.

| **TSP** | **Species** | **Accession number** |
| --- | --- | --- |
| CD63 antigen | *B. taurus* | NP_991372.1 |
| CD9 antigen | *B. taurus* | NP_776325.1 |
| Uroplakin-1b | *B. taurus* | NP_776907.2 |
| CD9 antigen | *C. sinensis* | GAA49954.1 |
| CD63 antigen | *D. rerio* | NP_955837.1 |
| CD81 antigen | *D. rerio* | NP_001003735.1 |
| Uroplakin-1a | *D. rerio* | NP_001035332.1 |
| CD63 antigen | *H. sapiens* | NP_001771.1 |
| CD81 antigen | *H. sapiens* | NP_004347.1 |
| Uroplakin-1b | *H. sapiens* | NP_008883.2 |
| CD63 antigen | *M. musculus* | NP_031679.1 |
| CD9 antigen | *M. musculus* | NP_031683.1 |
| Uroplakin-1b | *M. musculus* | NP_849255.2 |
| *Ov*-TSP-2 | *O. viverrini* | JQ678707.1 |
| *Ov*-TSP-3 | *O. viverrini* | JQ678708.1 |
| *Ov*-TSP-3 | *O. viverrini* | JQ678706.1 |
| MS3_02232 | *S. haematobium* | XP_035588046.1 |
| MS3_07569 | *S. haematobium* | XP_012798921.2 |
| MS3_08458 | *S. haematobium* | XP_012799761.1 |
| MS3_01905 | *S. haematobium* | XP_012793489.1 |
| MS3_03452 | *S. haematobium* | KAF1337732.1 |
| MS3_03944 | *S. haematobium* | XP_035590131.1 |
| MS3_01557 | *S. haematobium* | XP_035585741.1 |
| MS3_01094 | *S. haematobium* | XP_035585283.1 |
| MS3_03883 | *S. haematobium* | XP_012795408.1 |
| MS3_09789 | *S. haematobium* | XP_012801047.1 |
| MS3_09698 | *S. haematobium* | XP_035587525.1 |
| *Sh*-TSP-2 | *S. haematobium* | MK238557 |
| *Sh*-TSP-4 | *S. haematobium* | XP_012792774.1 |
| *Sh*-TSP-5 | *S. haematobium* | XP_012796668.1 |
| *Sh*-TSP-6 | *S. haematobium* | XP_012792980.1 |
| *Sh*-TSP-18 | *S. haematobium* | XP_012796731.1 |
| *Sh*-TSP-23 | *S. haematobium* | XP_012800477.1 |
| CD63 antigen | *S. japonicum* | CAX70616.1 |
| CD9/CD37/CD6 | *S. japonicum* | CAX70118.1 |
| *Sj*-TSP-1 | *S. japonicum* | AAW26928.1 |
| *Sj*-TSP-2 | *S. japonicum* | AAW24822.1 |
| *Sj*-TSP-3 | *S. japonicum* | AAW24863.1 |
| *Sj*-TSP-4 | *S. japonicum* | AAP05954.1 |
| *Sj*-TSP-5 | *S. japonicum* | AAW27174.1 |
| *Sj*-TSP-6 | *S. japonicum* | AAW26326.1 |
| *Sm*-TSP-2 | *S. mansoni* | AAN17276.1 |
| *Sm*-TSP-1 | *S. mansoni* | XP_002580456.1 |
| Tspan-1 | *S. mansoni* | XP_002577444.1 |
| Tetraspanin D76 | *S. mansoni* | XP_002575497.1 |
| *Sm*23 | *S.mansoni* | AAA73525.1 |
| Tetraspanin 18 | *S. mansoni* | XP_018649476.1 |
| CD63 antigen-like | *S.mansoni* | XP_018650438.1 |
| Putative tetraspanin | *S. mansoni* | XP_018653608 |
