# Supplementary material for: Characterisation of tetraspanins from Schistosoma haematobium and evaluation of their potential as novel diagnostic markers
Source: PLoS Negl Trop Dis. 2022 Jan 24;16(1):e0010151. doi: 10.1371/journal.pntd.0010151 (PMC8812969; doi:10.1371/journal.pntd.0010151)
Supplement: S3 Table — (DOCX) [file pntd.0010151.s007.docx]

**Supplementary Table S3.** Codes and suggested names for *Schistosoma haematobium* tetraspanins reported. Names have been assigned to match previously identified *Schistosoma mansoni* homologs. In the absence of a previously described homolog, names have been numerically assigned.

| ***S. haematobium* genome code** | **Suggested name** | ***S. mansoni* homolog GenBank Accession** | **Description** |
| --- | --- | --- | --- |
| MS3_01153 | *Sh*-TSP-4 | [KAF1337732.1](https://www.ncbi.nlm.nih.gov/protein/KAF1337732.1?report=genbank&log$=prottop&blast_rank=1&RID=VP8XBP7A016) | putative tetraspanin-CD63 receptor |
| MS3_09198 | *Sh*-TSP-23 | [AAA73525.1](https://www.ncbi.nlm.nih.gov/protein/AAA73525.1?report=genbank&log$=prottop&blast_rank=2&RID=VP9RPH7H01R) | Sm23 |
| MS3_05226 | *Sh*-TSP-5 | [XP_018645751.1](https://www.ncbi.nlm.nih.gov/protein/XP_018645751.1?report=genbank&log$=prottop&blast_rank=3&RID=VP9PYYES013) | tetraspanin, putative |
| MS3_05289 | *Sh*-TSP-18 | [XP_018649476.1](https://www.ncbi.nlm.nih.gov/protein/XP_018649476.1?report=genbank&log$=prottop&blast_rank=13&RID=VP9SPZ6D01R) | putative tetraspanin 18, isoform 1 |
| MS3_01370 | *Sh*-TSP-6 | [XP_018650438.1](https://www.ncbi.nlm.nih.gov/protein/XP_018650438.1?report=genbank&log$=prottop&blast_rank=3&RID=VP8XWCHC013) | cd63 antigen-like |
| *Sh*-TSP-2 | *Sh*-TSP-2 | [AAN17276.1](https://www.ncbi.nlm.nih.gov/protein/AAN17276.1?report=genbank&log$=prottop&blast_rank=2&RID=VP9X1VCU016) | *Sm*-TSP-2 |
