# Supplementary material for: Characterisation of tetraspanins from Schistosoma haematobium and evaluation of their potential as novel diagnostic markers
Source: PLoS Negl Trop Dis. 2022 Jan 24;16(1):e0010151. doi: 10.1371/journal.pntd.0010151 (PMC8812969; doi:10.1371/journal.pntd.0010151)
Supplement: S4 Table — (DOCX) [file pntd.0010151.s008.docx]

**Supplementary Table S4.** Lists of oligonucleotide primers flanking the LEL region of *Schistosoma haematobium* tetraspanins.

| TSPs | Forward primers | Reverse primers |
| --- | --- | --- |
| *Sh*-tsp- 2 | Ncol-Ndel-F 5´CGCCCATGGGTCATATGGAAAAGCCAAAGGTAAAAAAA3´ | Xho1-R-5´CGCCTCGAGGGTGCATTTTGCTTAGATCAC3´ |
| *Sh*-*tsp*-*4* | Ncol-Ndel-F -5´CGCCCATGGGTCATATGTCTCGTAAAGATGAGATTGGC3´ | Xho1-R-5´CGCCTCGAGATAATCCATATATTTAAGGAA3´ |
| *Sh*-*tsp*-*5* | Ncol-Ndel-F 5´CGCCCATGGGTCATATGAGAAAACAAGTCCCTCATACA3´ | Xho1-R-5´CGCCTCGAGTTGATGTAATGGTTTCAAGCA3´ |
| *Sh*-*tsp*-*6* | Ncol-Ndel-F 5´CGCCCATGGGTCATATGAGAGACGAAGTAAAATCTCAG3´ | Xho1-R-5´CGCCTCGAGCTTTTCAAAGAAGGAGATTAC3´ |
| *Sh*-*tsp*-*18* | Ncol-Ndel-F 5´CGCCCATGGGTCATATGGGCACACAAAGTTTGTGGAAG3´ | Xho1-R-5´CGCCTCGAGTGGAATAGCTATGAACGGGAG3´ |
| *Sh*-*tsp*-*23* | Ncol-Ndel-F 5´CGCCCATGGGTCATATGGTAGCAGTTGTTTACAAAGAT3´ | Xho1-R-5´CGCCTCGAGGTTGCGTTTCAAGAATGCTCC3´ |
