# Supplementary material for: Characterisation of tetraspanins from Schistosoma haematobium and evaluation of their potential as novel diagnostic markers
Source: PLoS Negl Trop Dis. 2022 Jan 24;16(1):e0010151. doi: 10.1371/journal.pntd.0010151 (PMC8812969; doi:10.1371/journal.pntd.0010151)
Supplement: Attachment — Submitted filename: Rebuttal_PNTD-D-21-01523.docx [file pntd.0010151.s009.docx]

30th July 2021

**Dear Prof Cantacessi and Dr Webster,**

REJOINDER: PNTD-D-21-01523

Entitled: Characterisation of tetraspanins from *Schistosoma haematobium* and evaluation of their potential as novel diagnostic markers.

We are very grateful for your time in considering our manuscript for publication at *PLoS Neglected Tropical Diseases* and your time and efforts in evaluating this manuscript.

All the points are very constructive and have contributed to enhance the quality of the manuscript. We believe that all the points have been addressed below.

Please, be also aware that we have included an extra author that should have been included in the previous version.

**Reviewer 1:**

We’d like to thank the reviewer for their positive comments about the study. They have no specific question or comment to address.

**Reviewer 2:**

We’d like to thank the reviewer for their positive comments about the study. They have no specific question or comment to address.

**Reviewer 3:**

We appreciate the reviewer’s positive comments, time, and effort to review the manuscript.

1. For ease of reading, I would recommend the authors name their new members of the TSP family (ShTSP1, 2 etc) instead of continuing to use the genome ID throughout. They have clearly shown in their supplementary figures that they are TSPs family members. I know this minor change will lead to quite a lot of changes in fugures and manuscript but it was hard to follow at times using the full gene codes.

*RESPONSE: We agree with the reviewer and have renamed the proteins based on homology to previously described S. mansoni tetraspanins. In the absence of a previously described homolog, and to avoid confusion with Sm-TSP-1, new names starting from Sh-TSP-4 have been assigned.*

2. I would also recommend the authors make it more explicit in the results about their prior publication, as in the results section it is unclear why the three TSPs are chosen to proceed to diagnostics based on the results shown. The prior mouse data didn’t clearly support the decision as MS3_05289 was not recognised nor were they linked in expression or location. I thought originally it was as the were all insoluble in protein production, but the answer mentioned in discussion is that the others were previously already examined. I think some noting of this in the results would aid in clarity.

*RESPONSE: We have included a sentence highlighting the reason of investigating diagnostic efficacy only for Sh-TSP-4, Sh-TSP-5 and Sh-TSP-18 in the results section (lines 402-405). Despite the low recognition by mouse serum, we also included Sh-TSP-18 in the study to include all members of the uroplakin family since all members of the CD63 family were studied in that prior publication.*

3. Phylogenetics

- methods section didnt seem to convey enough information relating to number of bootstraps, substitution model - it is needed for future understanding of the paper.

- figure legend for figure 1 is too limited - should at least mention the type of phylogenetic programme.

- results coverage may also have noted bootstrap support levels for the clustering as it enhances the arguement

*RESPONSE: We have added all information requested in lines 216-218; 353-359 and 834-835. Please, note that we have slightly modified some parts of the phylogenetic results since we found some minor mistakes.*

4. qPCR -method section states the use of 2 delta delta CT but from my understanding this assumes a uniform PCR amplification efficiency of 100%, but I didnt see any proof that this was the case for the transcrpts assessed.

*RESPONSE: Reviewer is right; however, we do not check routinely for PCR efficiency and, in our study and for a particular gene (and primers), we assume that efficiency is 100%. This method has been acceptable for many other publications in PLoS journals (see* [*https://doi.org/10.1371/journal.ppat.1008213*](https://doi.org/10.1371/journal.ppat.1008213)*) and other parasitology journals (see* [*https://doi.org/10.1007/s00436-018-6063-8*](https://doi.org/10.1007/s00436-018-6063-8) *or* [*https://doi.org/10.1186/s13071-019-3672-8*](https://doi.org/10.1186/s13071-019-3672-8)*).*

5. Minor aspects
   a. Table S1 S3 - use the correct prime symbol

*RESPONSE: Done*

b. Line 66 – slightly unclear as a “single female adult worm” wouldn’t release those level of eggs as it would surely require the worm to be paired with a male.

*RESPONSE: We appreciate the comment and have modified the sentence as follows: “… a single adult female worm, when paired, can release hundreds…”*

c. L143 – first “ml” needs a space between value and unit.

*RESPONSE: Done*

d. L436 – reference 58 is in a different form of brackets and is unlikely to be actually reference 58 as this is an S. mansoni paper. Maybe reference 38?

*RESPONSE: Reviewer is right, thanks for noticing. Reference has been modified.*

e. Supplementary Figure 1 and 2 legend - needed a bit more explanation - what do the colours represent?

*RESPONSE: Clustalx colouring scheme was applied. This has been added to the figure legend. This is an emulation of the default colourscheme used for alignments in Clustal X, a graphical interface for the ClustalW multiple sequence alignment program. Each residue in the alignment is assigned a colour if the amino acid profile of the alignment at that position meets some minimum criteria specific for the residue type.*

f. Supplementary Figure 3 legend- some specifics on expected size of recombinant proteins would have helped.

*RESPONSE: This information has been added in the Supplementary Figure 3 legend*

g. L881 - spelling of coomassie.

*RESPONSE: Thanks, fixed.*

**Reviewer 3:**

1. Line 157: The parentheses around reference 38 appears different from the other references.

*RESPONSE: Thanks for noticing. The reference has been modified.*

2. Line 163: volume written as ml it more frequently appears in the text as mL, authors should be consistent.

*RESPONSE: We changed all “ml” to “mL”*

3. Line 174: no manufacturer is listed for the Superscript III

*RESPONSE: Done*

4. In the discussion, the authors mention that MS3_09198 and 01370 have already been shown to be the targets of IgG responses in the serum and urine of S. haematobium positive individuals. Initially, I questioned why these two targets weren't included in the studies presented in this manuscript, as they appear to elicit the most robust responses in figure 4. As it is currently written in this manuscript, it's not really clear why MS3_09198 and 01370 are included since they aren't investigated in the human urine or AUC analysis.

*RESPONSE: A similar comment was also raised by Reviewer 3. We have included a few sentences clarifying this in lines 402-405.*

5. After reading the results I left wondering whether there was a relationship between the actual egg counts in the human urine samples and the ELISA OD values. I'm not sure if this information is available beyond the more general high, medium and low classifications used, but it would be interesting (if possible) to compare ELISA values to actual egg counts and to directly compare ELISA values between the three tests. Do the urine samples with the highest egg counts result in the highest ELISA values and is this consistent across the three targets?

*RESPONSE: We appreciate the reviewer’s comment. We actually did that analysis, but did not include the data since we saw no correlation between egg count and OD values (see Fig. 1 at the end of this rebuttal letter). We have included a line highlighting this lack of correlation (lines 415-416).*

6. A nitpicking detail related to Figure 5. Panel B is the only ELISA that uses a different scale than the other panels. For comparison purposes it would be helpful to unify the Y-axis of panel B to match the others.

*RESPONSE: We agree and thank the reviewer for noticing. Modified.*

7. I may have missed it, but I didnt come across a reference to Figure 5E in the results section.

*RESPONSE: Added.*

We hope it now meets the requirements for publication at *PLoS Neglected Tropical Diseases* and look forward to hearing from you.

Yours sincerely,

Javier Sotillo,

On behalf of all authors.

**Figure 1.** OD values observed for each protein tested (A-C) and the combination (D) in relation to the egg counts for each individual patient. No statistical correlation was observed.
